# Digital Specimen Tracking- and ISO 15189-Oriented Risk Management in Anatomic Pathology: A Qualitative Study of Expert Perspectives in Western Austria

**DOI:** 10.3390/diagnostics16060949

**Published:** 2026-03-23

**Authors:** Pius Sommeregger, Natalie Pallua, Bettina Zelger, Riem Kahlil, Johannes Dominikus Pallua

**Affiliations:** 1 MBA Risiko- und Krisenmanagment, Hochschule Burgenland, Campus 1, 7000 Eisenstadt, Austria; piussommeregger@gmail.com (P.S.); riem.khalil@amc.or.at (R.K.); 2 Department of Pediatrics, Innsbruck Medical University, Anichstraße 35, 6020 Innsbruck, Austria; natalie.pallua@tirol-kliniken.at; 3 Institute of Pathology, Neuropathology, and Molecular Pathology, Medical University of Innsbruck, Muellerstrasse 44, 6020 Innsbruck, Austria; bettina.zelger@i-med.ac.at; 4 Department of Orthopaedics and Traumatology, Medical University Innsbruck, Anichstraße 35, 6020 Innsbruck, Austria

**Keywords:** anatomic pathology, specimen tracking, traceability, ISO 15189, quality indicators, risk management, barcoding, RFID, qualitative research

## Abstract

**Background**: Breakpoints in the pre-examination processes and at organizational interfaces are a significant source of failures in specimen identification and tracking in anatomic pathology. While ISO 15189 emphasizes end-to-end traceability and risk-based quality management, implementing these principles in complex, multi-actor specimen pathways remains challenging. This study explores expert perspectives on specimen process chains, tracking mechanisms, and ISO 15189-oriented quality and risk management in pathology. **Methods**: We conducted 10 semi-structured expert interviews across three settings. Interviews were audio-recorded, transcribed, pseudonymized, and analyzed using structured qualitative content analysis (Mayring) supported by MAXQDA. A deductive category system derived from the theoretical framework and interview guide comprised six main categories and twelve subcategories. **Results**: Across 512 coded text segments, participants identified several factors as critical for effective implementation, including: (i) interface management along the specimen pathway, with recurrent vulnerabilities at handovers between operating theater/ward/transport and accessioning; (ii) the central role of barcode-based identification and the need for closed-loop traceability; (iii) the importance of measurable quality indicators and incident learning systems to operationalize risk management; (iv) persistent paper–digital handoffs and heterogeneous IT landscapes that undermine data integrity; (v) the need for clearly assigned responsibilities, training, and SOP governance; and (vi) implementation barriers including resources, change management, and vendor integration, alongside practical enablers such as incremental roll-out and cross-professional governance. **Conclusions**: Experts converge on a pragmatic ISO 15189-aligned roadmap: prioritize interface risks, standardize identifiers and handover rules, define a minimal KPI set for tracking and misidentification events, and reduce paper–digital handoffs by interoperable IT. Future work should quantify baseline error rates and evaluate the impact of digital tracking interventions on patient safety and turnaround times.

## 1. Introduction

Accurate specimen identification and traceability are fundamental to the reliability of diagnostic medicine. In anatomic pathology, each specimen represents an irreplaceable diagnostic substrate; any labeling or identification error can lead to a cascade of adverse outcomes ranging from delayed reporting to incorrect treatment or patient harm [1,2,3]. Despite advances in automation and informatics, extensive benchmarking studies by the College of American Pathologists and others have shown that specimen identification and labeling errors remain among the most frequent quality events in diagnostic laboratories [2,4]. These events are particularly prevalent in the pre-examination processes, where interfaces between collection, transport, accessioning, and processing introduce multiple opportunities for human error [5].

Historically, laboratory quality assurance relied heavily on retrospective audits and error tallies. However, the 2012 and 2022 revisions of the International Organization for Standardization (ISO) 15189 (Medical laboratories—Requirements for quality and competence) introduced a shift toward proactive, risk-based thinking [6]. ISO 15189 positions medical laboratories within a broader healthcare ecosystem that must continuously evaluate and control risks affecting patient safety and diagnostic validity. Under this framework, specimen traceability and information management are explicitly recognized as key determinants of process competence and overall quality [7].

In addition, ISO/TS 23824:2024 provides anatomic pathology-specific guidance for the application of ISO 15189 requirements to AP workflows, including end-to-end traceability, information integrity, and governance across multi-actor specimen pathways. In this study, we therefore use ISO 15189 as the primary normative anchor and explicitly relate our findings to ISO/TS 23824:2024 to support accreditation-oriented interpretation and implementation in anatomic pathology [8,9,10]. Implementation of ISO 15189 has demonstrated measurable benefits—including standardized documentation, improved staff awareness, and reduced examination error —but it also entails substantial organizational and documentation effort [6]. Laboratories frequently report barriers such as limited personnel, incomplete digital infrastructure, and parallel analog documentation systems, which can, paradoxically, generate new error pathways [6].

Digital specimen-tracking systems, particularly barcode-enabled identification and laboratory information system (LIS) integration, have been proposed as practical tools to operationalize ISO 15189 traceability requirements. Barcode-enabled identification and closed-loop tracking, particularly when combined with electronic order entry and verification, have been shown to substantially reduce specimen labeling and identification errors in real-world implementations and systematic evaluations [11,12,13,14].

However, heterogeneous IT environments and incomplete interoperability between hospital information systems (HIS) and LIS platforms often lead to “paper–digital handoffs,” where manual transcription or duplication reintroduces risk [15]. Furthermore, barcode systems alone cannot address upstream process issues such as missing clinical information or ambiguous specimen submission. Thus, the success of digital traceability depends on comprehensive process governance, staff training, and continuous monitoring through quality indicators.

Under ISO 15189, laboratories are required to establish measurable quality indicators (QIs) that reflect performance and patient safety risk. Common QIs include specimen rejection rates, labeling non-conformities, and turnaround-time deviations [7]. Yet many laboratories lack standardized frameworks for selecting, measuring, and interpreting these indicators within pathology workflows [16,17]. Qualitative evidence describing how professionals perceive these risk points and integrate ISO 15189 principles into routine operations remains scarce, particularly for anatomic pathology, which involves complex multi-step specimen handling and diverse professional roles [6,18,19,20,21,22]. Accordingly, expert interviews can provide high-yield process knowledge across interfaces and governance layers that are difficult to capture through event recording alone, particularly in heterogeneous IT and multi-site settings.

To address this knowledge gap, the present study explores how professionals across the pathology specimen pathway conceptualize traceability, risk management, and ISO 15189 implementation. Given the qualitative expert-interview design and the limited number of interviews, the study aims to generate in-depth, implementation-oriented insights rather than quantitative prevalence estimates. To support transparent interpretation, we explicitly report interview distribution across professional categories and institutional contexts and relate the resulting themes to ISO/TS 23824:2024 (Medical laboratories—Guidance on application of ISO 15189 in anatomic pathology) guidance domains [23]. Through ten expert interviews encompassing pathology, biomedical technicians, quality management, and IT/LIS roles, this study aims to:

- Identify process steps and interfaces perceived as risk hotspots for specimen identification and tracking.
- Examine how digital tracking and ISO 15189-aligned risk management practices are currently implemented and experienced;
- Derive pragmatic recommendations for enhancing traceability, reducing errors, and balancing documentation burden with diagnostic safety.

**Figure 1:**
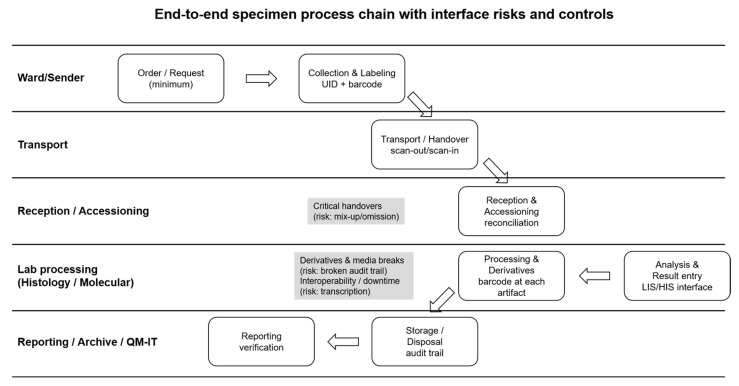
End-to-end specimen process chain with critical interfaces, identity controls, and implementation levers (ISO 15189 context). The swimlane process map summarizes the workflow from order/request and collection/labeling through transport, reception/accessioning, processing of derivatives, diagnostic examination/result entry, reporting, and archiving/disposal. Critical handovers and paper–digital handoffs are highlighted as points of elevated mix-up/omission and transcription risk. The figure also indicates key control measures and operational levers identified in the qualitative synthesis, including a unique identifier with barcode use across all artifacts, closed-loop scanning with reconciliation and documented exception handling, LIS/HIS integration with downtime procedures, and governance elements such as RACI-defined responsibilities, a minimal set of quality indicators with CAPA triggers, and structured training and audit routines.

By providing in-depth qualitative insights from multiple organizational levels, this study contributes to the evidence base required for designing risk-aware, digitally integrated, and sustainable quality-management systems in diagnostic pathology. Figure 1 summarizes the end-to-end specimen process chain, the key interface risks discussed by participants, and the corresponding operational controls and implementation levers. In line with ISO 15189:2022 and contemporary laboratory terminology literature, this study uses the terms pre-examination, examination, and post-examination to distinguish workflow stages from methodological or risk-related data analysis [24,25].

## 2. Materials and Methods

### 2.1. Study Design and Rationale

This study adopted a qualitative, multiple-case design to explore how professionals across the pathology specimen pathway conceptualize and operationalize traceability, quality, and risk management in accordance with ISO 15189 requirements.

An exploratory, interpretive approach was selected to capture context-dependent knowledge and to enable a nuanced understanding of organizational interfaces and safety culture. The research followed recognized frameworks for qualitative health services research—COREQ (Consolidated Criteria for Reporting Qualitative Research) and SRQR (Standards for Reporting Qualitative Research)—to ensure transparency, rigor, and reproducibility in study conduct and reporting [26,27].

### 2.2. Theoretical Background and Conceptual Framework

The conceptual framework combined key elements of risk and quality management in laboratory medicine with process-oriented models of traceability and system safety. ISO 15189:2022 (Medical laboratories—Requirements for quality and competence) served as the primary normative anchor, linking requirements for specimen identification, pre-examination integrity, and information management to broader quality objectives. Complementary models were selected because they are among the most widely used and practically applicable approaches in healthcare and laboratory quality management and because they offer complementary perspectives on system performance. Specifically, PDCA/PDSA was used as the overarching logic for continuous improvement, FMEA for structured prospective identification of failure modes and risk points, and Lean/Six Sigma to examine workflow inefficiencies, waste, process variation, and implementation barriers. Together, these approaches provided a pragmatic framework for identifying, organizing, and interpreting quality- and safety-relevant indicators across the laboratory system [28,29]. This framework guided both the design of the interview guide and the subsequent deductive category system used for data analysis.

### 2.3. Sampling and Participants

Participants were purposefully selected to ensure maximum variation in roles, institutional settings, and professional experience relevant to specimen tracking and quality management. Participant characteristics are summarized in Table 1.

Inclusion criteria were as follows:

1. At least three years of professional experience working in pathology, working as a biomedical technician, or working in laboratory quality management or LIS/IT administration;
2. Direct involvement in workflows related to specimen identification, traceability, or accreditation processes;
3. Employment within Austrian hospital or university pathology departments.

Recruitment followed a purposive snowball sampling strategy through professional networks and institutional contacts. The final sample comprised 10 experts: 3 pathologists (including 1 laboratory director), 3 biomedical technicians, 2 quality managers, and 2 IT/LIS specialists.

Participants represented three institutional contexts: a university pathology department, an extensive public hospital network, and a private pathology practice. Because this was a small multi-site qualitative study, accreditation status is reported only at an aggregated level to preserve institutional anonymity; participants were recruited from settings with experience in ISO 15189-oriented quality management and accreditation-related processes. To preserve institutional anonymity, specific LIS/HIS product names are not reported; accordingly, the digital environment is described at a functional level focused on interoperability, traceability, and workflow integration. Sampling continued until theoretical saturation was achieved. Saturation was assessed iteratively during data collection and analysis and was considered to have been reached when successive interviews no longer yielded new themes, subthemes, or conceptually relevant additions to the coding framework, and when additional interviews did not meaningfully extend the thematic understanding relevant to the study aims.

### 2.4. Data Collection

Data were collected via semi-structured expert interviews conducted between September and October 2025. An interview guide (Appendix A) was developed based on the theoretical framework and covered six thematic domains:

1. Process flow and interfaces;
2. Specimen identification and traceability;
3. Risk management and quality indicators;
4. Digitalization and LIS/HIS integration;
5. Organizational structure and training;
6. Implementation barriers and enablers.

Each interview lasted between 14 min 19 s and 42 min 37 s (median 22:52). Interviews were conducted in German via Microsoft Teams or in-person, recorded with informed consent, and subsequently transcribed verbatim. All personal identifiers were removed to preserve confidentiality. Field notes were written immediately after each session to document contextual factors and reflexive observations. Formal member checking was not performed as a transcript-return procedure for all participants. However, where organizationally feasible, central findings were reflected in condensed form with selected interview partners to support interpretive credibility.

### 2.5. Data Analysis

Data were analyzed using Mayring’s deductive qualitative content analysis to identify and structure key concepts relevant to ISO 15189-oriented traceability and risk management in anatomic pathology [30,31]. The deductive category system was operationalized from the conceptual framework, with ISO 15189:2022 as the primary normative reference for traceability, process control, and risk-based quality management. PDCA/PDSA, FMEA, Lean, and Six Sigma were selected as complementary and widely used approaches in healthcare and laboratory quality management because, together, they allow quality indicators and workflow vulnerabilities to be examined from continuous-improvement, risk-based, efficiency, and process-variation perspectives. Within this framework, PDCA/PDSA served as the overarching logic for continuous improvement, FMEA terminology structured the discussion of failure modes, causes, effects, and controls, and Lean/Six Sigma concepts were used to capture waste, variation, and implementation barriers. A deductive–inductive hybrid approach was applied. Deductive coding followed a predefined category system derived from the interview guide and theoretical framework (T1–T6 main categories and 12 subcategories). Inductive subcodes were added when new concepts emerged that were not adequately represented in the initial coding framework.

The analysis proceeded in three stages. First, all transcripts were initially coded in MAXQDA 24 by one researcher. Second, cross-coding verification by a second reviewer was used to enhance reliability; selected transcript passages were independently double-coded for calibration, discrepancies were discussed in consensus meetings, and the codebook was refined iteratively. Formal intercoder agreement was not quantified using Cohen’s kappa or a similar coefficient, as the aim was interpretive calibration and codebook refinement rather than statistical agreement testing. Third, thematic aggregation and frequency mapping were performed across all ten interviews. Final coding was conducted using stabilized code definitions and documented in an audit trail.

Coding frequencies were analyzed descriptively to illustrate relative emphasis across interviews; multiple rounds of coding per segment was allowed, reflecting the interconnected nature of the topics. Given the qualitative design and limited sample size, these frequencies are not intended as prevalence estimates of errors or failure modes. Saturation was monitored alongside codebook development and iterative analysis and was considered to be supported by stabilization of the category structure, such that later interviews no longer yielded new main themes, subthemes, or conceptually relevant additions to the coding framework.

### 2.6. Researcher Reflexivity

The lead researcher is a healthcare professional with prior experience in pathology quality management and risk analysis. Reflexive journaling and peer discussions were used to minimize interpretative bias and ensure that pre-existing professional knowledge informed (but did not dominate) data interpretation. A methodological overview is presented in Table 2. These frameworks informed both the interview guide and the deductive codebook used for analysis (Appendix A).

## 3. Results

Participants (*n* = 10) represented key stakeholder perspectives along the anatomic pathology specimen pathway, including three pathologists, three biomedical technicians, three quality managers, and two IT/LIS specialists, spanning three institutional contexts (university medicine, a public hospital network, and a community/private pathology practice). Interviews were conducted between September and October 2025 in German (on-site, phone, or Teams) and ranged from 14:19 to 42:37 min (median 22:52). Table 1 provides the anonymized interview distribution, role perspectives, interview mode, and duration to support transparent appraisal of the empirical basis of the qualitative analysis.

**Figure 2:**
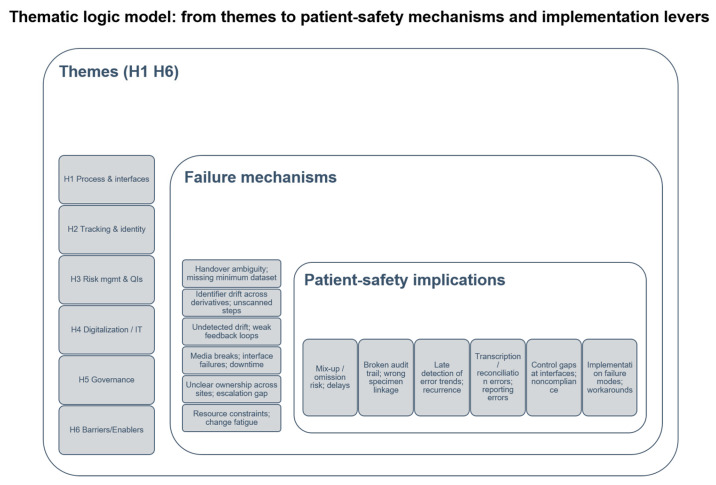
A thematic logic model linking qualitative themes to patient-safety mechanisms and actionable implementation levers. The model synthesizes the six higher-order themes (T1–T6) and connects typical failure mechanisms (e.g., handover ambiguity, identity drift across derivatives, paper–digital handoffs, weak feedback loops) to patient-safety implications (mix-up/omission risk, broken audit trails, transcription/reconciliation errors, delayed detection of process drift). Corresponding operational levers are mapped to each theme, including end-to-end process mapping with RACI-defined responsibilities, closed-loop traceability (unique identifier and barcode scanning), a minimal quality-indicator set with CAPA triggers, LIS/HIS integration with downtime procedures, formal governance and competency management, and phased implementation supported by leadership and change management.

Qualitative content analysis (Mayring, a deductive–inductive hybrid) generated six higher-order themes (T1–T6) and 12 subcategories (S1.1–S6.2). The themes map onto the study’s framework: ISO 15189 traceability requirements (identity continuity, documentation/auditability), PDCA-oriented improvement mechanisms, FMEA-style risk reasoning (failure modes and controls), and Lean/Six Sigma perspectives on process flow, waste and variation. In total, 512 meaningful text segments (sentences or short paragraphs) were coded and distributed across these subcategories. Segment counts and approximate shares are reported to convey relative emphasis across interviews; because multiple coding per segment was permitted, percentages should be interpreted as descriptive indicators of salience rather than mutually exclusive prevalence measures. The most frequent subcategories were risk assessment and measures planning (S3.2; 65; ~13%), specimen reception/early processing (S1.1; 50; ~10%), deviations/error sources (S3.1; 46; ~9%), implementation barriers (S6.1; 46; ~9%), IT/LIS quality and interfaces (S4.1; 43; ~8%), and labeling/traceability (S2.1; 38; ~7%) (Table 3). Coding frequencies for the most frequent subcategories are summarized in Table 3, while the end-to-end process perspective and cross-theme implementation logic are summarized in Figure 1 and Figure 2, respectively.

To translate the qualitative themes into an implementation-oriented framework, we developed a logic model linking themes to failure mechanisms, patient-safety implications, and operational levers (Figure 2).

### 3.1. Specimen Process Chain and Interface Risks (T1)

T1 captures expert statements describing the end-to-end specimen process chain in anatomic pathology and the risk concentration at interfaces between steps and professional groups. In total, T1 comprised 86 coded segments across two subcategories: S1.1, specimen reception/early processing (50 segments; ~10%), and S1.2, critical handover points (36 segments; ~7%). Participants consistently emphasized that safety-critical failures rarely originate in a single isolated step; instead, they emerge when responsibilities, information, and specimen identity need to be “carried” across transitions (Figure 1).

Reception and early processing (S1.1) was repeatedly framed as a high-leverage control point because it is the first point at which the laboratory can verify whether the incoming specimen package is internally consistent and complete. Experts described systematic checks at entry as essential to prevent downstream rework and avoid propagation of errors into derivative artifacts (cassettes, blocks, slides, and molecular aliquots). A representative quote illustrates this gatekeeping role: “I work in specimen reception; we must ensure everything is complete—request form, label, specimen.” Operationally, participants linked this to the need for standardized entry criteria, including a minimum dataset and explicit rules for handling incompleteness.

Critical handovers (S1.2) were described as the dominant source of latent risk, particularly where the process crosses organizational or professional boundaries. Participants highlighted that the probability of mix-ups, omissions, and delays increases when (i) ownership is unclear (“who is accountable at the interface?”), (ii) information must be manually transcribed or re-entered, or (iii) artifacts are relabeled, split, pooled, or re-packaged. These interface vulnerabilities were not limited to the pre-examination processes; rather, participants also pointed to risk at derivative creation and routing steps, where identity must remain consistent across multiple physical and digital representations of the same case.

Across both subcategories, experts converged on the need to translate process knowledge into implementable controls: a shared end-to-end process map, explicit handover rules (including what must be checked and documented at each transition), and clearly assigned responsibilities. The patient-safety implications and corresponding implementation levers derived from T1 are consolidated in Table 4, while the workflow structure and interface points are visualized in Figure 1.

### 3.2. Tracking and Identity Assurance (T2)

T2 aggregates expert statements on specimen identification, labeling, and traceability across the pathology workflow, with a particular focus on whether identity is maintained consistently from the primary container to all derivative artifacts (e.g., cassettes, blocks, slides, molecular aliquots) and the corresponding digital records. T2 comprised 64 coded segments across two subcategories: S2.1 labeling/traceability (38 segments; ~7%) and S2.2 errors and loss events (26 segments; ~5%). Across interviews, participants treated identity assurance as the core precondition for patient safety and diagnostic validity: once traceability is broken, subsequent technical excellence cannot reliably compensate.

Labeling and traceability practices (S2.1) were described as varying in maturity and consistency, particularly at points where a specimen is split, repacked, relabeled, or otherwise transformed into derivatives. Several experts highlighted “closed-loop” approaches—typically involving barcode-supported checks and role-separated verification—as the operational backbone of reliable traceability. One representative quote illustrates the desired target state: “Everything is checked with four eyes; it is transparent and traceable who did what.” In these descriptions, “traceable” did not merely mean “label present,” but rather a documented, system-supported history of who performed which action at which time and under which case identifier, ideally anchored in the LIS.

Errors and loss events (S2.2) were reported as occurring less as single catastrophic failures and more as minor disruptions of the audit trail that accumulate: missing or ambiguous labels, mismatches between request form and container, non-scanned status changes, temporary “workarounds” during peak workload, and re-identification steps not mirrored in the information system. Participants emphasized that the highest-risk situations occur when the physical artifact and the digital record drift apart (e.g., manual re-entry of identifiers, copying identifiers from paper, or creating derivatives before the case is fully accessioned). In such cases, the audit trail may remain partially reconstructable, but the reconstruction effort itself introduces additional opportunities for error and delays, and the residual uncertainty can persist into reporting.

Operationally, experts converge on a small set of measures that make identity assurance robust across the entire chain (Figure 1): (i) a unique identifier assigned early and carried consistently across all derivatives; (ii) barcode-based scanning at every status-relevant transition (receipt, accessioning, grossing, embedding, microtomy, staining, distribution, reporting, archiving); (iii) reconciliation steps when artifacts are split/merged or when deviations occur; and (iv) documented exception handling (what triggers an incident record, who is notified, when work must stop, and how the case is cleared for continuation). The patient-safety implications and actionable implementation levers derived from T2 are summarized in Table 4, and the points where identity assurance is most vulnerable (handover interfaces and derivative creation) are visualized in Figure 1.

### 3.3. Risk Management and Quality Indicators (T3)

**Figure 3:**
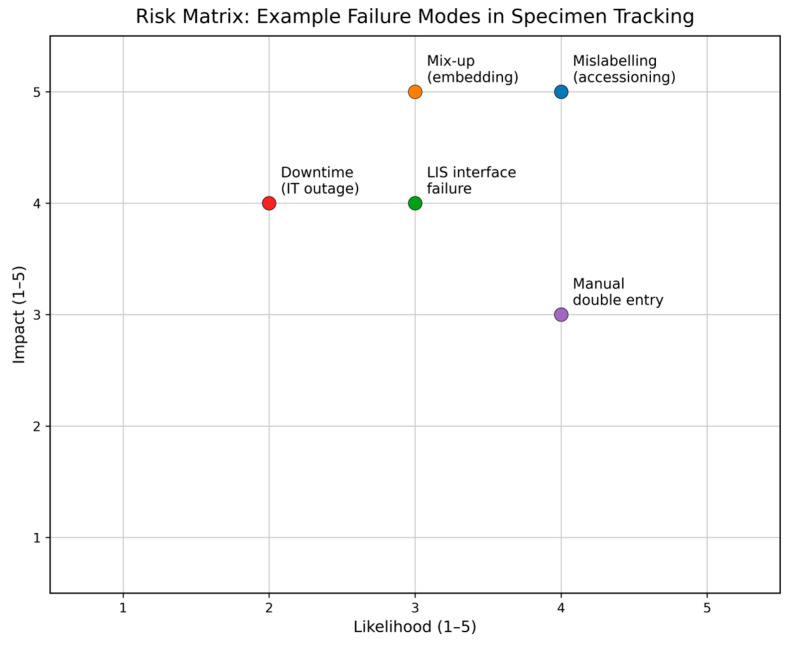
Example risk matrix for specimen tracking failure modes. Likelihood (1–5) is plotted on the *x*-axis and impact/severity (1–5) on the *y*-axis. Points indicate representative failure modes across key workflow steps (e.g., accessioning, embedding, IT/LIS interface, and documentation), shown for illustration of prioritization within an ISO 15189-oriented risk management framework.

T3 captures expert statements on how laboratories identify, document, and mitigate risks in the specimen pathway and how they use quality indicators (QIs) and CAPA mechanisms to detect process drift and prevent recurrence. T3 was the most prominent theme in the dataset, comprising 111 coded segments across two subcategories: S3.1 error sources and deviations (46 segments; ~9%) and S3.2 risk assessment and mitigation (65 segments; ~13%). Participants consistently described risk management as most effective when it was operationalized as a closed feedback loop: deviations were recognized early, analyzed consistently, translated into corrective/preventive actions, and monitored via a small set of indicators. To illustrate how a risk assessment matrix can be operationalized for specimen tracking, representative failure modes were mapped to a 1–5 likelihood and 1–5 impact scale (Figure 3).

Error sources and deviations (S3.1) were described across pre-examination, examination, and post-examination steps, but with a recurring emphasis on interface-driven failures—particularly where manual transcription, incomplete request information, or ambiguous ownership at handovers creates latent vulnerability. Participants noted that deviations are frequently detected “late” (e.g., during reconciliation, when a derivative is missing, or at sign-out), which increases rework and heightens the probability of downstream consequences. Importantly, experts did not frame deviations as exceptional events; rather, they were described as predictable failure modes that require systematic learning rather than ad hoc fixes.

Risk assessment and mitigation (S3.2) contained the largest share of coded segments and included practical descriptions of how ISO-aligned risk work is actually carried out (or where it remains aspirational). A representative quote illustrates the “learning system” approach participants aimed for: “We document errors in quality management and check whether we can redesign the process so it won’t happen again.” In these accounts, effective mitigation depended less on the formal existence of procedures and more on whether there were clearly defined triggers that turned an observed issue into structured CAPA work (including root-cause analysis, responsibility assignment, implementation, and effectiveness checks).

Across interviews, experts repeatedly highlighted that QIs and CAPAs are often not standardized across teams or sites, making it challenging to compare performance, identify early systemic drift, or ensure that improvement actions are sustained. Participants therefore advocated for a minimal, harmonized QI set that focuses on identity and interface integrity rather than exhaustive measurement. Examples of operationally meaningful indicators described by participants included completeness of minimum dataset at reception, frequency of identifier mismatches or relabeling events, number of unscanned transitions or reconciliation events, turnaround time disruptions attributable to pre-analytical issues, and rates of CAPA recurrence for the same failure mode. A common theme was that QIs should be tied to explicit escalation thresholds (CAPA triggers) and mapped to defined owners (governance/RACI) so that corrective actions do not depend on informal individual initiative.

Overall, T3 indicates that ISO 15189 risk management is most actionable when it is translated into routine workflows: structured incident learning, reproducible CAPA pathways, and a small number of indicators that function as an early-warning system for drift. The patient-safety implications and implementation levers derived from T3 are consolidated in Table 4, and their integration into an implementation-oriented framework is reflected in Figure 2.

### 3.4. Digitalization, IT/LIS Infrastructure, and Paper–Digital Handoffs (T4)

T4 summarizes expert statements on how digital infrastructure and interoperability shape patient safety in the pathology specimen pathway. The theme comprises 69 coded segments across two subcategories: S4.1 system quality and interfaces (43 segments; ~8%) and S4.2 paper–digital handoffs and data integrity (26 segments; ~5%). Across interviews, participants consistently connected digitalization to safety outcomes through two mechanisms: (i) the extent to which LIS/HIS enable reliable identity continuity and traceability, and (ii) the degree to which “paper–digital handoffs” require manual transcription and reconciliation work that increases error probability.

System quality and interfaces (S4.1) covered the perceived reliability of LIS/HIS environments, interface functionality across departments and sites, and the usability of systems in high-throughput routine operations. Experts described interoperability limitations as a recurrent driver of workarounds (e.g., duplicate documentation, manual copy/paste of identifiers, parallel lists), particularly at interfaces between wards/OR and the laboratory and in multi-site settings. Several participants framed robust LIS/HIS interfacing not as an IT convenience but as a patient-safety control, because it reduces ambiguity regarding what is ordered, what has been received, what derivatives exist, and where each artifact is located within the process chain (Figure 1).

Paper–digital handoffs and data integrity issues (S4.2) were repeatedly described as safety-critical because they introduce manual handling steps precisely where identity assurance must remain stable. One representative quote captures the perceived mechanism: “If something is handwritten and then digitized and copied, there are naturally sources of error.” Participants noted that paper–digital handoffs can occur not only when paper forms are used, but also when digital systems are not integrated (e.g., partial interfaces, temporary local solutions, or systems that do not exchange identifiers and status information reliably). Experts described how these breaks can lead to transcription errors, mismatches between physical artifacts and digital records, delayed reconciliation, and—in extreme cases—uncertainty about case completeness or provenance.

A recurrent operational topic was downtime handling. Participants emphasized that IT failures and planned maintenance are predictable events and require explicit procedures to prevent the emergence of uncontrolled workarounds. In these accounts, downtime procedures were considered adequate only when they included clear rules for temporary documentation, unambiguous linkage of temporary identifiers to the master identifier, reconciliation steps upon system recovery, and defined responsibility for clearance to resume routine workflows. Participants also mentioned the need for system-level validation checks (e.g., plausibility checks for identifiers, prevention of duplicate case creation, and enforcement of scan events at key status transitions), which were described as mechanisms to reduce reliance on individual vigilance.

Overall, T4 indicates that digitalization contributes to safety by reducing manual transcription, maintaining identity continuity across systems, and providing robust fallback procedures during downtime. The resulting patient-safety implications and implementation levers are consolidated in Table 4; their relationship to interface vulnerabilities and the cross-theme implementation logic is reflected in Figure 1 and Figure 2.

### 3.5. Organizational Structure, Responsibilities, and Training (T5)

T5 aggregates expert statements describing how organizational structures, role clarity, and staff qualification influence the reliability of the specimen pathway, particularly at interfaces and in multi-site settings. The theme comprises 55 coded segments across two subcategories: S5.1 roles and responsibility (19 segments; ~4%) and S5.2 qualification and training (36 segments; ~7%). Across interviews, participants emphasized that even well-designed technical controls (e.g., barcode tracking, LIS integration) remain fragile if ownership is unclear, escalation pathways are not standardized, or staffing and competency management do not match process complexity.

Roles and responsibilities (S5.1) focused on the practical question of “who owns the interface” when responsibility crosses professional or institutional boundaries. Participants described control gaps as most likely when responsibility is distributed but not explicitly governed—particularly in situations involving multiple locations, rotating teams, or parallel workflows. Experts also highlighted that ambiguity at interfaces can lead to informal practices that vary by shift or individual, which makes both error prevention and post hoc reconstruction more difficult. In these accounts, clarifying ownership was not framed as an administrative exercise but as a prerequisite for effective incident handling, because deviation management depends on knowing who must decide, who must act, and who must be informed at each transition.

Qualification and training (S5.2) were described as a decisive enabler of safe routine operations and sustainable implementation of ISO-aligned practices. Participants repeatedly noted that training needs extend beyond onboarding and include periodic refreshers, training after process changes (e.g., new interfaces, new barcode rules, LIS updates), and structured competence assessment. A representative quote conveys both the perceived importance and the underlying resource dependency: “Training, training, training—most important is having enough people and resources to train.” Participants described that training-related vulnerabilities become particularly visible during workload peaks, staffing shortages, or implementation phases, when untrained staff may rely on informal workarounds or “shadow systems,” increasing variability and the probability of identity or documentation errors.

Operationally, T5 points to several recurring implementation requirements: (i) explicit responsibility assignment and interface governance (e.g., RACI-style role definition for key steps and transitions); (ii) standardized escalation pathways for incomplete documentation, identifier mismatches, missing derivatives, and downtime-related reconciliation; and (iii) structured competency management, including role-specific training curricula, key-user concepts for LIS/barcode workflows, and documented authorization for safety-critical tasks. Participants also linked governance and training to audit readiness and learning capacity, noting that consistent responsibilities and documented competence facilitate both internal quality work and external accreditation-related assessments.

Overall, T5 demonstrates that organizational clarity and competency management function as cross-cutting controls that stabilize technical and procedural measures across the specimen process chain. The patient-safety implications and actionable levers derived from T5 are consolidated in Table 4, and their integration into the implementation logic is reflected in Figure 2.

### 3.6. Implementation Barriers and Enablers (T6)

T6 summarizes expert statements on practical conditions that hinder or facilitate the implementation of risk-based, ISO 15189-aligned specimen-tracking and governance measures in routine pathology operations. The theme comprises 81 coded segments across two subcategories: S6.1 barriers (46 segments; ~9%) and S6.2 enablers (35 segments; ~7%). Across interviews, participants described implementation success as dependent on how well technical solutions and procedural requirements align with workload realities, staffing, and change capacity, and on whether documentation requirements are perceived as delivering a proportional safety benefit.

Barriers (S6.1) were described primarily as resource and complexity constraints rather than resistance in principle. Participants repeatedly reported that time pressure, staffing shortages, and high throughput can incentivize informal workarounds, particularly when new controls (e.g., scanning requirements, additional checks, and expanded documentation) are perceived as adding steps without immediate visible benefit. A representative quote captures this tension in accredited environments: “In an accredited area, you have to document a lot—I feel like I fill out 100 checklists a day.” Experts noted that excessive or redundant documentation can become counterproductive by diverting attention from safety-relevant checks to form completion and by encouraging “checkbox compliance” rather than meaningful risk control. Barriers were also linked to heterogeneity across sites (different systems, differing local routines), partial interoperability, and unclear governance—conditions that increase implementation variability and complicate standardization.

Enablers (S6.2) were most often framed as organizational and managerial conditions that allow safe practices to be adopted and sustained. Participants described leadership support as critical, particularly when it translates into protected time for training, realistic staffing plans during rollout phases, and clear prioritization of safety-relevant process changes. Experts also noted that acceptance improves when implementation is phased, starting with high-risk interface points (e.g., accessioning and derivative creation) and expanding once workflows stabilize. Several participants emphasized the importance of simplifying and harmonizing SOPs and forms, reducing local variation, and ensuring that tools are usable in practice (e.g., scanners available at the point of work, interfaces that minimize re-entry, and clear downtime procedures). In these accounts, enablers also included transparent communication of the “why” (patient safety and audit-trail integrity), feedback loops that demonstrate measurable improvement, and visible responsiveness to user-reported issues during implementation.

Operationally, T6 suggests that implementation planning should explicitly address failure modes that arise during change itself. Participants advocated approaches that balance documentation load with safety value, define realistic milestones, and embed monitoring early so that drift and workaround behavior can be detected and corrected. In addition, they highlighted the need to align governance and competency management with implementation: clear ownership and escalation pathways reduce ambiguity during rollout, while structured training and key-user support improve adherence and reduce variability across shifts and sites.

Overall, T6 illustrates that implementation is most robust when it is treated as a managed, risk-aware change process rather than a one-time technical deployment. The patient-safety implications and actionable levers derived from T6 are consolidated in Table 4, and their integration with the cross-theme implementation logic is summarized in Figure 2.

## 4. Discussion

This qualitative study explored expert perspectives on end-to-end specimen tracking and ISO 15189-aligned quality and risk management in anatomic pathology. Interpreting these findings through the combined ISO 15189/PDCA/FMEA/Lean Six Sigma framework (Table 2) highlights that traceability failures arise from (a) discontinuities in identity documentation and audit trails (ISO 15189), (b) weak closed-loop improvement routines (PDCA), (c) recurrent and predictable failure modes at interfaces and during downtime (FMEA logic), and (d) workflow waste and variation driven by hybrid documentation and local workarounds (Lean/Six Sigma).

Across 512 coded segments, six dominant themes emerged: (i) risk concentration at process interfaces; (ii) partial but inconsistent digital traceability; (iii) variability in risk management and quality-indicator practice; (iv) recurrent “paper–digital handoffs” in hybrid digital–analog workflows; (v) the pivotal role of training and governance; and (vi) the operational burden of documentation in accredited settings. Given the qualitative expert-interview design (*n* = 10; median 22:52 min), these findings should be interpreted as context-anchored perspectives rather than quantitative prevalence estimates. These themes can also be read against ISO/TS 23824:2024, particularly its anatomic pathology–specific emphasis on end-to-end traceability, information integrity, interface governance, and controlled handling of specimen derivatives across the workflow. Segment counts and approximate shares (Table 3; top six subcategories) are reported to describe the relative salience of topics raised by participants rather than statistical prevalence. Because coding permitted multiple assignments per text segment and the analytic unit is not independent in a statistical sense, percentages should be interpreted as descriptive signals of emphasis within this dataset, not as population estimates. Accordingly, frequency differences are used here to prioritize implementation-relevant risk points, while transferability relies on the transparency of context and workflow description rather than on quantitative inference.

Collectively, these findings indicate that specimen identification and traceability remain system-level safety challenges rather than purely technical problems, consistent with prior evidence that most identification errors originate in the pre-examination phase and at organizational handovers [1,2,3,32].

Our results reinforce earlier large-scale surveys showing that misidentification and mislabeling events persist even in laboratories with mature quality systems [2,3,4]. Participants’ emphasis on pre-examination vulnerabilities aligns with published evidence from the total testing process, where pre-examination (including extra-laboratory) steps have been reported to account for a substantial proportion of mistakes—up to ~70% in some analyses [5,33,34].

Consistent with prior work on barcode-enabled traceability, experts recognized digital identification as the most tangible safeguard, yet noted gaps in interoperability and coverage. In ISO 15189 terms, this translates into maintaining an uninterrupted identity chain and an auditable event history across all specimen derivatives and handovers. Studies implementing barcode systems across the specimen chain—particularly when paired with electronic order entry—report substantial reductions in labeling and identification errors, as supported by both implementation studies and systematic evidence [11,12,13,15,35]. However, partial digitization without integration can introduce new failure modes, a phenomenon mirrored in participants’ reports of paper–digital handoffs and parallel paper tracking.

Interoperability and planned or unplanned system downtimes should be treated as safety-critical controls, not merely IT reliability issues. Evidence from patient-safety event reports shows that EHR downtime introduces concrete hazards—particularly around patient identification and communication of clinical information—because electronic safeguards (e.g., automated verification, decision support, standardized routing) are partially or fully deactivated [36,37]. In laboratory and pathology contexts, LIS downtime or LIS–EHR interface failures similarly disrupt result reporting and increase reliance on manual transcription and ad hoc workarounds; published downtime approaches emphasize predefined contingency workflows, reconciliation steps, and rapid restoration of the electronic audit trail [38,39]. From an FMEA perspective, downtime-related workarounds constitute a high-severity failure mode because they simultaneously reduce detectability (missing electronic checks) and increase occurrence (manual re-entry), making predefined controls and post-downtime reconciliation essential risk mitigation strategies. This aligns closely with participants’ accounts in T4, which indicate that paper–digital handoffs and downtime periods amplify identity and traceability risks, underscoring the need for tested downtime SOPs, explicit responsibility for “go/no-go” decisions, and structured post-downtime reconciliation. More broadly, interoperability itself has been associated with patient-safety and quality outcomes in the EHR literature, while studies of clinical laboratories show persistent variation and generally limited capability to exchange information electronically—conditions that can perpetuate manual re-entry and reconciliation work [40,41]. The perceived duality of ISO 15189—which is conceptually valuable but resource-intensive—echoes international analyses of accreditation roll-outs. Laboratories frequently cite limited personnel, financial constraints, and documentation overload as the main obstacles [6,9]. This mirrors a Lean/Six Sigma tension: controls add value when they prevent defects and stabilize flow, but become waste when documentation is redundant, poorly integrated, or not tied to feedback loops. Despite these challenges, accreditation has been shown to improve error detection, process consistency, and staff awareness [7,42].

Participants’ focus on risk management and incident learning also aligns with the literature on Failure Mode and Effects Analysis (FMEA) in clinical laboratories, which effectively prioritizes high-severity failure modes [28,29,43]. Nonetheless, the experts in this study highlighted that such analyses are rarely embedded into everyday routine, emphasizing the need for operationalized, pragmatic risk-indicator dashboards rather than episodic audits. Crucially, participants described effectiveness not as the presence of a risk tool per se, but as the existence of a functioning PDCA routine: indicators and deviations (Check) trigger structured CAPA work (Act/Do), and effectiveness checks close the loop (Plan/Check).

Operationalized through the ISO 15189/PDCA/FMEA/Lean Six Sigma lens (Table 2), three practical implications emerge:

1. Traceability Depth and Coverage: Barcode or RFID identifiers should extend to all specimen derivatives (cassettes, blocks, slides) and be logged at each handover, ensuring longitudinal traceability. The absence of a digital event record at any step can compromise the entire audit trail [13,14].
2. Interoperable Information Infrastructure: True digital safety gains depend on LIS–HIS integration, standardized order entry, and downtime protocols that prevent information loss during outages. As several participants observed, hybrid paper steps reintroduce transcription risks [15,37,38].
3. Risk-Aware Governance and Quality Indicators: ISO 15189: 2022 now mandates proactive risk management and measurable indicators for key processes. Our findings support a minimal, high-impact set of indicators—such as a mislabeled specimen rate, reconciliation mismatches, and rework frequency—that can drive continual improvement without an excessive reporting burden [7].

Implementing these measures aligns with the growing emphasis on diagnostic stewardship and on integrating patient-safety principles across laboratory and pathology workflows [44,45,46]. A key strength of this study is its multi-perspective design, including clinicians, pathologists, biomedical technicians, and IT/QM specialists, thereby capturing both technical and organizational dimensions of traceability. The use of deductive content analysis guided by ISO 15189 provides theoretical coherence, while anonymized verbatim quotations preserve authenticity. Several limitations should be considered when interpreting these findings. First, the study is based on a relatively small qualitative sample of ten expert interviews across three institutional contexts, with short-to-moderate interview durations. Although this design was appropriate for generating implementation-relevant, in-depth perspectives across interfaces, it limits breadth and does not support statistical generalizability or prevalence estimates. In addition, some professional and institutional perspectives were represented by only one or a few participants, which may have left certain local practices or dissenting views underrepresented. Accordingly, coded-segment counts and approximate shares are presented only as descriptive indicators of salience within this dataset. Second, the findings reflect the Austrian healthcare and pathology context and should therefore be interpreted as context-anchored expert perspectives with limited transferability to other countries, health systems, or organizational settings. Third, the study relies on self-reported expert accounts, which may be influenced by selective recall, social-desirability bias, and partial visibility of process failures. Finally, because no direct observational or quantitative process data were included, the findings cannot be directly correlated with measured error frequencies or system performance metrics. Future research should link qualitative insights with quantitative error metrics and turnaround-time data to evaluate the effect of digital tracking and risk-management maturity on diagnostic safety outcomes. Longitudinal implementation studies, ideally using before-and-after or stepped-wedge designs, could assess how ISO 15189-aligned digital traceability influences measurable patient-safety and efficiency indicators. Additionally, integration of AI-assisted monitoring (e.g., automated label verification, anomaly detection in LIS data streams) offers potential for proactive risk prediction—extending the principles identified in this study toward a learning digital pathology system.

## 5. Conclusions

This qualitative expert interview study indicates that specimen identification and traceability in anatomic pathology are primarily shaped by system-level interface risks rather than by isolated technical failures. Participants consistently identified vulnerability in the pre-examination processes and at handovers between senders, transport, and accessioning, where incomplete information, inconsistent responsibilities, and hybrid workflows can compromise downstream diagnostic reliability.

Experts viewed barcode-enabled traceability and standardized operating procedures as key safeguards, but emphasized that safety gains depend on end-to-end coverage (including derivatives such as cassettes, blocks, and slides) and on interoperable IT/LIS infrastructures that minimize paper–digital handoffs and support reliable event logging, reconciliation, and downtime procedures. Risk management was considered highly relevant yet variably formalized, underscoring the need to operationalize ISO 15189–aligned governance through pragmatic, risk-based prioritization and a minimal, high-impact set of quality indicators linked to CAPA processes.

Overall, the findings support a pragmatic implementation roadmap: (i) prioritize high-risk interfaces, (ii) standardize identifiers and traceability checkpoints across the specimen lifecycle, (iii) reduce analog–digital transitions through integration and robust fallback procedures, and (iv) invest in training and role clarity to sustain safe practice beyond audit-driven compliance. Future work should complement these qualitative insights with quantitative tracking metrics and prospective evaluations of digital traceability interventions on error rates, turnaround times, and patient-safety outcomes.

## Figures and Tables

**Table 1:** Participant overview (anonymized, condensed from EI1–EI10). Abbreviation: EI—expert interview participant identifier.

Participant	Primary Role Perspective (Anonymized)	Interview Mode	Duration (min:s)
EI1	Data quality/management; prior lab leadership perspective	On-site	24:29
EI2	OR/ICU interface; data quality/coding	On-site	14:19
EI3	Academic physician–scientist; interface to (molecular) pathology workflow	On-site	29:40
EI4	Pathology leadership	On-site	33:33
EI5	Sending-site physician perspective	On-site	20:45
EI6	Biomedical technician perspective	On-site	21:15
EI7	Pathologist perspective	Phone	42:37
EI8	Lead biomedical technician’s perspective	On-site	26:44
EI9	Biomedical technician/laboratory perspective	On-site	18:44
EI10	Biomedical technician’s perspective	On-site	14:48

**Table 2:** Methodological overview.

Parameter	Description
Design	Qualitative, multiple-case exploratory design
Approach	Semi-structured expert interviews; Mayring content analysis
Participants	*n* = 10 (pathologists, biomedical technicians, QM and IT specialists)
Framework	ISO 15189, PDCA, FMEA, Lean Six Sigma
Data Collection	September–October 2025; Teams/in-person; 14–43 min per interview
Data Analysis Tool	MAXQDA 24
Validation	Cross-coding, COREQ adherence, peer debriefing
Ethics	Informed consent; institutional waiver; GDPR compliance

**Table 3:** Top six most frequent subcategories across all interviews (coded segments; N = 512 total coded segments). The complete subcategory frequency distribution is provided in Appendix A (Table A1).

Subcategory	Topic	Coded Segments (n)	Approx. Share
S3.2	Risk assessment and measures planning	65	~13%
S1.1	Specimen receipt and early processing	50	~10%
S3.1	Deviations, error sources	46	~9%
S6.1	Implementation barriers	46	~9%
S4.1	IT/LIS quality and interfaces	43	~8%
S2.1	Labeling and traceability	38	~7%

**Table 4:** Summary of themes and actionable implications.

Theme	Subcategories (Coded Segments; Share)	Representative Quote (Translated)	Typical Issue Described by Experts	Patient-Safety Implication	Actionable/Operational Levers
T1 Process chain and interfaces	S1.1 Reception/processing (50; ~10%); S1.2 Critical handovers (36; ~7%)	“I work in specimen reception; we must ensure everything is complete—request form, label, specimen.”	Many handovers; unclear ownership	Higher mix-up/omission risk	End-to-end process map; RACI responsibilities; standardized entry criteria; explicit handover rules (minimum dataset, accountability at transitions)
T2 Tracking and identity assurance	S2.1 Labeling/traceability (38; ~7%); S2.2 Errors/loss (26; ~5%)	“Everything is checked with four eyes; it is transparent and traceable who did what.”	Traceability varies across derivatives	Breaks the audit trail	Unique identifier + barcode at every artifact; closed-loop traceability (barcode scanning at status changes); reconciliation; documented exception handling
T3 Risk management and quality indicators	S3.1 Error sources (46; ~9%); S3.2 risk assessment/mitigation (65; ~13%)	“We document errors in quality management and check whether we can redesign the process so it won’t happen again.”	QIs/CAPA not standardized	Late detection of drift	Minimal QI set; CAPA triggers; operationalize ISO 15189 risk work via incident learning; CAPA workflows; small KPI set to track performance.
T4 Digitalization/IT (LIS/HIS) and data integrity	S4.1 System quality/interfaces (43; ~8%); S4.2 paper–digital handoffs/integrity (26; ~5%)	“If something is handwritten and then digitized and copied, there are naturally sources of error.”	Paper–digital handoffs; limited interoperability	Transcription and reconciliation errors	LIS/HIS integration; reduce paper steps; downtime SOPs; validation checks for identifiers; strengthen interoperability and data-quality controls
T5 Governance, responsibilities and training	S5.1 Roles/responsibility (19; ~4%); S5.2 Qualification/training (36; ~7%)	“Training, training, training—most important is having enough people and resources to train.”	Responsibility unclear across sites	Control gaps at interfaces	Formal governance; audits; training; clarify ownership and escalation pathways; competency management (onboarding, refreshers, key-user model)
T6 Implementation barriers and enablers	S6.1 Barriers (46; ~9%); S6.2 Enablers (35; ~7%)	“In an accredited area you have to document a lot—I feel like I fill out 100 checklists a day.”	Resources + change management challenges	Implementation failure modes	Phased rollout; leadership support; balance documentation load with safety value; streamline SOPs/forms; secure resources and change-management capacity

## Data Availability

The data presented in this study are available upon request from the corresponding author.

## Abbreviations

The following abbreviations are used in this manuscript:

AI	Artificial Intelligence.
CAPA	Corrective and Preventive Action.
COREQ	Consolidated Criteria for Reporting Qualitative Research.
FMEA	Failure Mode and Effects Analysis.
HIS	Hospital Information System.
ISO 15189	International Standard for Medical Laboratories—Requirements for Quality and Competence.
IT	Information Technology.
KPI	Key Performance Indicator.
LIS	Laboratory Information System.
QIs	Quality Indicators.
RACI	Responsible–Accountable–Consulted–Informed (responsibility matrix).
RAM	Risk Assessment Matrix.
SOP	Standard Operating Procedure.
SRQR	Standards for Reporting Qualitative Research.

## Appendix A. Coding Framework (Deductive Category System) and Operational Definitions

For reliability checking, an independent second reviewer double-coded a subset of material for calibration; disagreements were resolved through discussion and used to refine operational definitions before final coding. Formal intercoder agreement was not quantified, as the procedure was intended to support interpretive consistency and codebook refinement.

B1. Overview

Interview transcripts were analyzed using a deductive qualitative content analysis approach. A priori categories were derived from the conceptual framework and the interview guide (Appendix A) and operationalized in a coding guide. The final code system comprised six main categories (T1–T6) and twelve subcategories (S1.1–S6.2). Coding was performed in MAXQDA; multiple codes could be applied to a text segment where appropriate.

B2. Codebook: Main Categories and Subcategories (Operational Definitions)

T1. Process Chain of Specimen Handling and Interface Risks

**Definition** **A1.**
*Statements describing the end-to-end specimen pathway, process steps, and risks at transitions/handover points (sender–transport–accessioning–laboratory workflow–reporting–archiving).*

Inclusion: Descriptions of workflow steps; identification of bottlenecks; handover responsibilities; examples of “critical transitions”.

Exclusion: Purely technical IT interface issues (code under T4) unless tied directly to a process handover.

- S1.1 Specimen reception and early processing
  Focus: receipt, registration, accessioning checks, completeness review, initial handling and routing.
- S1.2 Critical handover points
  Focus: vulnerable interfaces (collection → transport → reception; grossing → embedding; microtomy → staining; sign-out → communication).

T2. Specimen Tracking and Identity Assurance

**Definition** **A2.**
*Statements describing mechanisms that ensure correct specimen identity and traceability across all derivatives (container, cassette, block, slide) and along the workflow.*

Inclusion: barcode/RFID use, double-checks, audit trails, relabel rules, reconciliation, tracking events, traceability depth.

Exclusion: General training or governance statements not specifically about identity assurance (code under T5).

- S2.1 Labeling and traceability
  Focus: identifier standards, barcode workflows, labeling rules, traceability across derivatives.
- S2.2 Mix-ups, losses, and exception handling
  Focus: incidents (loss, mismatch, mislabeling), detection, escalation, resolution, documentation.

T3. Risk Management and Quality Indicators (ISO 15189-Oriented)

**Definition** **A3.**
*Statements describing risk identification, assessment, prioritization, mitigation, monitoring, and continuous improvement (including CAPA and quality indicators).*

Inclusion: FMEA/RAM use; risk prioritization (likelihood × severity/detectability); incident learning; KPIs/QIs; thresholds and triggers; CAPA.

Exclusion: IT system performance as such (code under T4) unless directly framed as risk control.

- S3.1 Deviations and error sources
  Focus: where/why deviations occur across pre-examination/examination/post-examination phases; typical consequences.
- S3.2 Risk assessment and measures planning
  Focus: structured risk methods, decision-making, action planning, evaluation of effectiveness.

T4. Digitalization, IT/LIS Infrastructure, and Data Integrity

**Definition** **A4.**
*Statements describing digital systems (HIS/LIS), interoperability, interface quality, paper–digital handoffs, and information governance influencing traceability and process reliability.*

Inclusion: order entry, interface failures, paper–digital handoffs, downtime workarounds, logging, dashboards/alerts, data integrity risks.

Exclusion: Purely process/role content without IT component (T1/T5).

- S4.1 System quality and interfaces
  Focus: LIS usability/performance, HIS–LIS integration, scanners, event logging, interface stability.
- S4.2 paper–digital handoffs and data integrity risks
  Focus: manual transcription, parallel documentation, paper lists/spreadsheets, outage fallback and audit trail gaps.

T5. Organizational Structure, Responsibilities, and Training

**Definition** **A5.**
*Statements describing governance, role clarity, competency management, training, and documentation practices enabling sustained quality and safety.*

Inclusion: RACI-like role demarcation, training strategies, competency matrices, SOP governance, audit readiness.

Exclusion: Risk methods and KPIs (T3) unless framed as organizational capability rather than content.

- S5.1 Roles and responsibilities
  Focus: accountability, escalation pathways, interface ownership, leadership responsibility.
- S5.2 Qualification, training, and documentation
  Focus: onboarding, refresher training, competence assessment, SOP adherence, documentation burden.

T6. Implementation of Tracking/Risk Management/ISO 15189

**Definition** **A6.**
*Statements describing adoption, rollout, and sustainability of tracking and ISO-related processes, including barriers, enablers, and lessons learned.*

Inclusion: resource constraints, change management, acceptance, leadership support, pilot rollouts, champions, “quick wins”.

Exclusion: Specific risk methods (T3) unless discussed as implementation challenge.

- S6.1 Barriers and challenges
  Focus: personnel/time constraints, IT interoperability, documentation load, transport/pre-examination complexity.
- S6.2 Enablers and lessons learned
  Focus: implementation strategies, governance models, training approaches, incremental rollouts, success factors.

B3. Coding Frequencies (Coded Segments)

Note: Counts reflect coded segments in the qualitative analysis summary; multiple coding per segment may occur depending on analytic settings.

**Table A1:** Distribution of coded segments across subcategories (S1.1–S6.2). The table summarizes the number of coded text segments assigned to each subcategory and their approximate share of the total coding output (N = 512), providing a descriptive indication of topic salience across interviews.

Subcategory	Description	Coded Segments (n)	Approx. Share
S3.2	Risk assessment and measures planning	65	~13%
S1.1	Specimen reception and early processing	50	~10%
S3.1	Deviations and error sources	46	~9%
S6.1	Implementation barriers	46	~9%
S4.1	IT/LIS quality and interfaces	43	~8%
S2.1	Labeling and traceability	38	~7%
S1.2	Critical handover points	36	~7%
S5.2	Qualification, training, documentation	36	~7%
S6.2	Enablers and lessons learned	35	~7%
S2.2	Mix-ups/losses/exception handling	26	~5%
S4.2	Paper–digital handoffs and data integrity risks	26	~5%
S5.1	Roles and responsibilities	19	~4%
Total coded segments		512	100%

B4. Coding Notes (for transparency)

- Unit of coding: meaningful text segments (sentences or short paragraphs).
- Coding strategy: deductive framework aligned with ISO 15189-oriented process control; optional inductive refinement within subcategories where needed.
- Overlap: where permitted, a segment could receive multiple codes (e.g., a handover issue caused by an IT media break).
- Audit trail: coding guide and coded segment summaries were maintained to support the traceability of analytic decisions.
